# Stress as a Potential Modifier of the Impact of Lead Levels on Blood Pressure: The Normative Aging Study

**DOI:** 10.1289/ehp.10002

**Published:** 2007-03-19

**Authors:** Junenette L. Peters, Laura Kubzansky, Eileen McNeely, Joel Schwartz, Avron Spiro, David Sparrow, Robert O. Wright, Huiling Nie, Howard Hu

**Affiliations:** 1 Department of Environmental Health and; 2 Department of Society, Human Development, and Health, Harvard School of Public Health, Boston, Massachusetts, USA; 3 Veterans Affairs Boston Healthcare System and Boston University Schools of Medicine and Public Health, Boston, Massachusetts, USA; 4 The Channing Laboratory, Department of Medicine, Brigham and Women’s Hospital, Harvard Medical School, Boston, Massachusetts, USA; 5 Department of Environmental Health Sciences, University of Michigan School of Public Health, Ann Arbor, Michigan, USA

**Keywords:** blood pressure, bone lead, hypertension, psychosocial stress

## Abstract

**Background:**

Lead exposure and psychological stress have been independently associated with hypertension in various populations, and animal studies suggest that when they co-occur, their effects may be exacerbated.

**Objectives:**

We examined whether psychological stress modifies the impact of cumulative lead exposure (measured as bone lead levels) on hypertension and blood pressure in Boston-area community–exposed men participating in the Normative Aging Study.

**Methods:**

We evaluated the modifying effect of stress on lead exposure on baseline hypertension status (513 participants) and on blood pressure in those without hypertension (237 participants), cross-sectionally. In baseline nonhypertensives, we examined the same risk factors in relation to prospective risk of developing hypertension.

**Results:**

Cross-sectional analysis revealed a positive interaction between stress and tibia lead on systolic blood pressure, after adjusting for age, body mass index, family history of high blood pressure, education, smoking, alcohol consumption, physical activity, and nutritional factors. In prospective multivariate analyses, high stress also modified the effect of tibia lead and patella lead on the risk of developing hypertension. Those reporting high stress had 2.66 [95% confidence interval (CI), 1.43–4.95] times the risk of developing hypertension per standard deviation increase in tibia lead and had 2.64 (95% CI, 1.42–4.92) times the risk per standard deviation increase in patella lead.

**Conclusion:**

To our knowledge, these are the first analyses to look at interactive effects of stress and lead on hypertension in humans. These results suggest that the effect of lead on hypertension is most pronounced among highly stressed individuals, independent of demographic and behavioral risk factors.

Hypertension or high blood pressure affects approximately one-third of the U.S. adult population (Fields et al. 2004) and is a leading risk factor for morbidity and mortality from sudden death, heart disease, congestive heart failure, stroke, and renal insufficiency. For > 90% of hypertension, the cause is unknown. It is suspected that multiple environmental, psychosocial, and genetic factors play a role in the disease and that these factors may act additively or interactively (Schwartz et al. 2003).

Previous studies have shown an association between biological markers of lead exposure and elevated blood pressure. Many of the studies that used blood lead levels (which reflect mostly recent exposure) showed stable effect estimates but inconsistent associations with blood pressure (Hertz-Picciotto and Croft 1993; Staessen et al. 1996); however, more recent studies that used bone lead levels [which reflect cumulative lead exposure and are assessed using K-shell X-ray florescence (KXRF) bone lead measurements] have shown more consistent associations with increased blood pressure and particularly with risk of hypertension (Cheng et al. 2001; Glenn et al. 2003; Hu et al. 1996; Korrick et al. 1999; Lee et al. 2001; Martin et al. 2006). Because approximately 95% of the lead in adults is stored in the bone, even with the significant decline in environmental lead exposure, the release from this bone store to blood and soft tissue can be an ongoing important source of exposure and toxicity, especially in older populations (Rabinowitz 1991; Silbergeld 1991; Silbergeld et al. 1993; Tsaih et al. 2001).

Psychological stress can be defined as a response to life events (stressors) that are perceived or appraised as taxing the individual’s ability to cope with the demands imposed. An individual’s perception of a situation as stressful is a pivotal component in the process whereby a stressor affects health (Cohen et al. 1997; Lazarus and Folkman 1984; Monroe and Kelley 1997). Previous studies have shown psychosocial factors including stressful tasks, psychological distress, occupational stressors, and social alienation to be associated with elevated blood pressure in both laboratory and cohort studies (e.g., Kaplan and Nunes 2003; Levenstein et al. 2001). Some cohort studies have also suggested that stress or distress may play a role in the development of hypertension (e.g., Davidson et al. 2000; Jonas and Lando 2000; Jonas et al. 1997; Levenstein et al. 2001; Markovitz et al. 1993; Rutledge and Hogan 2002; Spiro et al. 1995).

The mechanism by which self-reported stress and lead jointly contribute to hypertension is not well understood. Exposure to low levels of lead seems to cause interference with sodium transport, affect the renin–angiotensin–aldosterone system, stimulate the hypothalamic–pituitary axis, increase sympathetic activity and catecholamines, and elevate the level of reactive oxygen species (e.g., Gonick and Behari 2002; Schwartz 1991; Vaziri and Sica 2004). Stress is also thought to affect blood pressure via multiple mechanisms activating the hypothalamic–pituitary axis, the renin–angiotensin system, and the sympathetic nervous system (e.g., Black and Garbutt 2002), and through behavioral pathways as well.

An interactive effect between psychological stress and lead on blood pressure has been demonstrated in animal studies, where lead exposure was shown not only to produce a stress reaction (Vyskocil et al. 1990, 1991a, 1991b) but also to heighten the harmful impact of other types of stressful situations on the function of the hypothalamic–pituitary–adrenal axis (Virgolini et al. 2005) as well as on neurotransmission and behavior (Cory-Slechta et al. 2004).

We hypothesized that older men reporting high stress would have a steeper dose response to the effect of bone lead on baseline hypertension status and blood pressure and on subsequent risk of developing hypertension compared with subjects reporting low stress. To test these hypotheses, we evaluated interactions between stress perception and bone lead on baseline hypertension status, systolic blood pressure (SBP), and diastolic blood pressure (DBP) cross-sectionally, and on incidence of hypertension prospectively, in a sample of community-dwelling older men from the Normative Aging Study (NAS).

## Materials and Methods

This research was conducted on a subgroup of the participants in the NAS, a longitudinal study of aging established in 1963 by the Veterans Administration (now the Department of Veterans Affairs). The cohort and subgroup of participants used in this research have been described elsewhere (Cheng et al. 2001; Hu et al. 1996). Briefly, the NAS is a closed cohort of 2,280 male volunteers from the Greater Boston area. Men were screened at entry and enrolled if they had no history of chronic medical conditions. In addition, enrollment was restricted to those with SBP < 140 mmHg and DBP < 90 mmHg. Since enrollment, participants have been re-evaluated every 3–5 years using questionnaires and detailed onsite physical examinations.

Between 1991 and 1996, 797 participants had bone lead content measured by KXRF. Participants were also given a series of questionnaires including questions about stress perception between 1987 and 1993. KXRF and questionnaire measurements were matched for the same year; however, if no questionnaire measurement was available for that year, the questionnaire data in the evaluation cycle up to 3 years before were used. For this study, we defined hypertension as diagnosis of hypertension with treatment by the participant’s regular physician or SBP > 140 mmHg or DBP > 90 mmHg during the study clinic examination. We used two sets of outcomes: *a*) hypertensive status and blood pressure (in a subset without hypertension) at the time of the first bone lead measurement (baseline exam), and *b*) development of hypertension in subjects without hypertension at baseline. For the latter, we used a follow-up period through 31 December 2004.

This study complied with all applicable requirements of the U.S. regulations, including institutional review board approval and written informed consent from all participants before administering study protocol. This study was approved by the Human Research Committees of Brigham and Women’s Hospital and the Department of Veterans Affairs Boston Medical Center.

### Stress measure

To assess participant stress perception, we administered the Health and Social Behavior Questionnaire (Aldwin and Revenson 1987; Aldwin et al. 1996; Yancura et al. 2006). To anchor the stress response to a concrete experience, participants were asked to think of the most stressful thing that occurred to them in the past month. The problem could be major or minor, something that was resolved or ongoing. Types of problems reported related to health of self or others, marital and wife, children and grandchildren, social, work, retirement, financial, general hassles, retirement, and bereavement (Aldwin et al. 1996). They were then asked, compared with other problems in the past, how stressful this problem was (how much it bothered or troubled them) rated on a 7-point scale where 1 = “not troubled” and 7 = “the most troubled I’ve ever been.” This score provided our measure of self-reported stress, with higher numbers indicating a greater self-reported stress. To facilitate the interpretability of interactive effects, stress level was dichotomized based on a median split, following other work in this area (Ohman et al. 2007). Men with scores below the median (≤5) were categorized as low self-reported stress and those with scores above (> 5) as high self-reported stress.

Evidence of the validity of this measure can be found in a recent study of stress and coping, where this single item measure of stress was found to be positively associated with a sense of threat and negative affect, and negatively associated with a sense of challenge and positive affect (Yancura et al. 2006). To further demonstrate the validity of the self-reported stress measure for our study, it was compared (in continuous form) with a global distress index [General Severity Index (GSI)], and two negative affect subscales (depression and anxiety) derived from the Brief Symptom Inventory, a self-report measure of psychological and somatic distress, and with the Perceived Stress Scale (PSS; Cohen et al. 1983), available in a subset of the NAS sample. As expected, the scaled measure of self-reported stress was moderately correlated with GSI (*r* = 0.21; *p* < 0.01), the anxiety subscale (*r* = 0.17; *p* < 0.01), depression subscale (*r* = 0.21; *p* < 0.01), and PSS (*r* = 0.23; *p* < 0.01).

### Bone lead measurement

We measured bone lead for 30 min each at the mid-tibia shaft and patella using a KXRF instrument (ABIOMED, Inc., Danvers, MA). The tibia and patella have been used for bone lead research because they consist primarily of cortical and trabecular bone, respectively, with differing toxicity potential for each. Technical specifications and validity of this instrument are described in detail elsewhere (Burger et al. 1990; Hu et al. 1990, 1994).

### Statistics

The main objectives of this study were to evaluate whether stress affects the relationship between bone lead and *a*) baseline hypertension status, *b*) baseline SBP and DBP in nonhypertensives (both cross-sectional models) and *c*) the risk of developing hypertension (prospective model among participants not hypertensive at baseline).

Tibia and patella bone lead measurements with estimated uncertainties > 10 and 15 μg/g of bone, respectively, were excluded as part of our laboratory’s quality control procedures (Hu et al. 1996). In this study, six participants were excluded. These levels of uncertainties usually reflect excessive patient movement during measurement. If a subject had more than one bone lead or questionnaire measurement, the earliest corresponding measurements were used. Lead levels were modeled per standard deviation change in tibia lead (11.6 μg/g) and patella lead (17.1 μg/g). All analyses use the dichotomized version of self-reported stress unless otherwise stated.

#### Cross-sectional models

We used logistic regression (dichotomous hypertension outcome) and linear models (continuous blood pressure outcomes) to evaluate the interactive effect of perceived stress and bone lead at baseline. Covariates were chosen based on biology, other studies, and potential mediating effects and included age and age squared (Cheng et al. 2001); sodium, potassium, and calcium intake (milligrams per day) (Appel 2003; Cheng et al. 2001; Elmarsafawy et al. 2006); family history of hypertension; body mass index (BMI; kilograms per square meter); educational level (graduated high school vs. less than high school); pack-years of smoking; alcohol consumption (grams per day); and physical activity (kilocalories per week).

#### Prospective model

We used Cox proportional hazards models to assess the interaction of stress and bone lead on hypertension risk prospectively. The same confounders were included in the Cox proportional hazards models as in the cross-sectional models. We also tested the models to determine whether they satisfied the assumption of proportionality. The follow-up period was until a participant developed hypertension or 2004, whichever came first. All analyses were conducted using the Statistical Analysis System (Unix SAS version 8.2; SAS Institute Inc., Cary, NC). A *p*-value < 0.05 was considered significant and < 0.10 marginally significant.

## Results

### Sample characteristics

Of the initial group of 791 participants with valid bone lead measurement, 513 also completed the stress measures. Compared with those for whom stress was not assessed, those with stress assessment did not differ on age; BMI; family history of hypertension; alcohol consumption; pack-years of smoking; physical activity; sodium, potassium, calcium, and vitamin D intake; and DBP. Those without stress assessment had higher SBP and lower anxiety and educational level. The groups were comparable with respect to tibia and patella lead levels. The mean (± SD) age of the 513 participants was 66.9 ± 7.1 years. Mean blood pressure measures were, for SBP, 135 ± 17.0 mmHg, and for DBP, 81 ± 9.6 mmHg. Mean tibia lead was 21.5 ± 13.4 μg/g, and mean patella lead 31.5 ± 19.3 μg/g.

Of these 513 participants with both valid bone lead measurements and stress assessment, 276 had hypertension and 237 did not. Table 1 shows the characteristics for hypertensives and nonhypertensives. There was no apparent difference between those with and those without hypertension in likelihood of participating in the stress and coping questionnaire. Among the subjects without hypertension, compared with those who had no stress assessment, those who had did not differ on any variable except DBP (higher in those without stress measure).

### Combined effects of stress and lead on hypertension status and blood pressure: cross-sectional analyses

#### Hypertension status

When we controlled for confounders, neither the main effects of self-reported stress and bone lead (tibia lead or patella lead) nor the interactive terms of self-reported stress by bone lead were significant predictors of baseline hypertension status (Table 2). BMI, family history of hypertension, and sodium intake were significant predictors.

#### Blood pressure in subjects without hypertension

**Figure 1:**
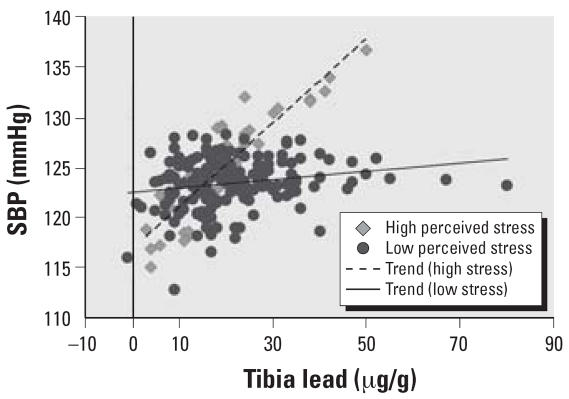
The relationship between tibia lead and estimated SBP for those with high self-reported stress versus those with low self-reported stress.

In the main effect models, controlling for confounders, self-reported stress was marginally significantly predictive [β = 2.59; 95% confidence interval (CI), −0.47 to 5.64] of SBP. In the adjusted tibia lead interaction model, self-reported stress was a marginally significant predictor of SBP, with those with high self-reported stress having an estimated increase in SBP of 2.89 mmHg (95% CI, −0.16 to 5.94) (Table 3). There was also a significant interaction between tibia lead and stress (β = 3.77; 95% CI, 0.46 to 7.09). The slope of SBP for each standard deviation increase of tibia lead for those with high stress was 3.57 (95% CI, 0.39 to 6.75) and for those with low stress −0.21 (95% CI, −1.70 to 1.29) (Figure 1). Neither tibia lead, self-reported stress, nor the interaction of stress and tibia lead was predictive of DBP.

In the adjusted patella lead interaction model, self-reported stress was a marginally significant predictor of SBP, with those with high stress having an estimated increased SBP of 2.98 mmHg (95% CI, −0.12 to 6.08) (Table 3). There was no significant interactive effect of patella lead with self-reported stress, although the direction of effects was similar to those seen with tibia lead. Self-reported stress was not predictive of DBP, and there was no significant interactive effect with patella lead.

To take better advantage of the information in the measure, we also examined the above relationships using the continuous form of the stress measure. Results were similar but of greater magnitude. In the main effect model, self-reported stress was significantly associated with systolic blood pressure (β = 0.95; CI, 0.12 to 1.78), in addition to being significant in the models with tibia and patella lead. The interaction with tibia lead on systolic blood pressure was also significant (β = 1.03; 95% CI, 0.24 to 1.82).

### Prospective association of combined effects of stress and lead on incident hypertension

Among the 237 subjects without hypertension but with reported stress perception, there were follow-up data for 220 men. The average years of follow-up was 6.2 ± 3.2 years, ranging between 2.5 and 13.0 years. Of the 220, 97 new cases of hypertension were observed during the follow-up period. In the longitudinal models, all the variables were found to be proportional.

Table 4 shows the results of the interactive models of bone lead and stress on incidence of hypertension. When we controlled for the same potential confounders as well as baseline SBP and DBP, none of the main effects were significant; however, there was an interactive effect for both the model with tibia lead and the model with patella lead. For the tibia lead model, those with high self-reported stress had 2.66 (95% CI, 1.43 to 4.95) times the risk of developing hypertension for each standard deviation increase of tibia lead than those with low stress. For the patella lead model, those with high self-reported stress had 2.64 (95% CI, 1.42 to 4.92) times the risk of developing hypertension for each standard deviation increase of patella lead than those with low stress. Similar to the cross-sectional models, the interactions between bone lead and self-reported stress were also observed when using the continuous form of the stress measure [for tibia, rate ratio (RR) = 1.30; 95% CI, 1.10 to 1.55; for patella, 1.23; 95% CI, 1.04 to 1.45]. Self-reported stress was also marginally significantly associated with development of hypertension in the main effect model (RR = 1.13; 95% CI, 0.98 to 1.31)

## Discussion

We examined cross-sectional and prospective effects of self-reported stress on the relationship between bone lead and hypertension. Our findings indicate that in this population of older men, bone lead is more likely to be associated with elevated SBP and with increased risk of developing hypertension among men with higher levels of self-reported stress than among those reporting lower stress levels. This lead–stress interaction is intriguing and consistent with findings in the animal literature, but to our knowledge this is the first time it has been reported in a human population.

Previous research has found separate relationships between stress and hypertension and lead and hypertension, but our research is among the first to suggest that they may jointly have specific and detrimental effects on cardiovascular health. Stress is suspected to modify the relationship between other environmental pollutants as well, and health outcomes such as atopic disease and ulcer (Shigemi et al. 1999; Wright 2005). For example, Shigemi et al. (1999) observed an interactive effect of perceived job stress on the relationship between the history of peptic ulcer and smoking.

Findings of a link among self-reported stress, lead, and SBP are particularly interesting given that SBP is directly and continuously related to the risk of stroke or coronary event, and is often included in algorithms developed for predicting the occurrence of cardiovascular disease. An effect of lead and stress was not seen on DBP, consistent with other literature that found effects of stress and of lead on only SBP (e.g., Cesana et al. 2003; Cheng et al. 2001). One reason may be that unlike SBP, which increases linearly with age, DBP increases with age up to age 55 and then declines thereafter (Burt et al. 1995; Izzo et al. 2000; Strandberg and Pitkala 2003). Because of this relationship of DBP with age, elevated DBP is more common in the young and middle-aged than in the elderly (Strandberg and Pitkala 2003). Alternatively, elevated SBP has become the most common form of hypertension in the U.S. aging population and is the more important parameter for determining risk of hypertension-related complications (Izzo et al. 2000; Strandberg and Pitkala 2003). As a result, we might expect SBP to be the more relevant parameter for our cohort, which has an average age of 66.9 ± 7.1.

We found no interactive effect between stress and bone lead in relation to whether a participant was hypertensive at baseline. Similar to other work, our definition of hypertension included both those who had doctor-diagnosed hypertension and those who were grouped as hypertensive based on high SBP or DBP during physical examination. Diagnosed hypertension means that participants are being treated and as a result may be changing behaviors and engaging in stress management. In this case, they may appear to have the same stress levels as those who are not hypertensive, making it difficult to see an effect of stress on prevalent hypertension.

For both patella and tibia lead, we observed the trend of higher blood pressure in the higher stress group; however, this relationship was significant only with tibia lead. Tibia lead is made up mostly of cortical bone and has slower turnover than patella bone, which is made up mostly of trabecular bone (Hu et al. 1991). The differences in effect in cross-sectional analyses may reflect a differential effect of mobilizable versus long-term lead stores; however, given the similarity in trends, the differences are more likely the result of the higher uncertainties in patella measurement (Cheng et al. 2001; Hu et al. 1991). This is additionally supported by the consistency between findings from the cross-sectional and prospective analyses. In the prospective analyses, there was a significant interaction with lead in both patella and tibia with stress on the prospective incidence of hypertension.

Regulatory and legislative efforts to reduce lead hazard in the United States beginning in the 1970s have resulted in a continued decline in blood lead in the adult population (approximately an 87% decrease between 1976–1980 and 1999–2002) (Muntner et al. 2005; Pirkle et al. 1994). However, in adults, most lead (approximately 95%) is stored in bone, and this lead has a long half-life of years to decades depending on the bone type. This means that older populations, such as our cohort, who have experienced decades of pre-1970 environmental exposure can have significant accumulation of lead in bone. The constant low-level interchange with soft tissue may then present a current source of toxicity, which, when coupled with exposure to stress, may put them at greater risk for hypertension.

Our results demonstrating an interactive relationship may be confounded by a neurologic effect of lead exposure on mood states and also stress perception (with more negative moods contributing to higher levels of perceived stress). In a study of occupationally exposed patients, integrated blood lead (which was used in the study as a measure of cumulative lead exposure) was related to general distress (Lindgren et al. 1999). Another study in the NAS cohort found a cross-sectional association between bone lead and phobic anxiety (Rhodes et al. 2003). However, in our prospective analysis, even among men with the same cumulative lead exposure, those who were experiencing higher stress were more likely to develop higher blood pressures, which would suggest that stress is modifying the effect of lead. Of note, this prospective analysis excluded hypertensives, and participants did not know their bone lead levels at the time of stress assessment; so it is unlikely that knowledge of blood pressure or bone lead influenced stress perception. Notwithstanding, this study does not address the issue of the timing of exposures that contribute to cumulative lead level, in terms of neurologic development. Therefore, men who were exposed earlier in life may have experienced more lasting physiologic changes that may have then made them more prone to judge experiences as distressful (Aldwin et al. 1989; Dohrenwend et al. 1984; Virgolini et al. 2005). Secondary analyses additionally controlling for the Brief Symptom Inventory global measure of psychological and somatic distress yielded little change (< 1% reduction) in the parameter estimates and no change in the significance of the effects in any of the models.

Significant relationships were observed controlling for a number of potential confounders: age, BMI, family history of hypertension, pack-years of smoking, alcohol consumption, physical activity, and nutritional factors; however, residual confounding remains possible, and other important variables may not have been considered. Furthermore, this study may have limited generalizability, being a male cohort that was 97% white with slightly higher than median income. Because lead exposure and stress appear to co-occur with low socioeconomic status, these findings may have greater import in these latter populations (Dohrenwend 1973; Virgolini et al. 2005). Previous analysis of this cohort has shown that those who did not participate in the bone lead substudy had comparable blood pressures and blood lead (Cheng et al. 2001). Moreover, both groups had comparable self-reported stress and levels of distress (i.e., anxiety). Thus, selection of participants for this substudy is unlikely to have been influenced by lead levels, stress levels, or risk of hypertension.

In conclusion, self-reported stress was found to modify the effect of lead on blood pressure and incident hypertension in a community sample of older men. Compared with those with lower levels of self-reported stress, among men with higher levels, there was a significantly stronger association between lead levels and SBP. Additionally, in prospective analysis, baseline self-reported stress modified the effect of baseline bone lead on the incidence of hypertension. With an increase in the prevalence of hypertension, the aging of generations with high community lead exposure and the potentially deleterious effect of hypertension on cardiovascular health, these findings may point to intervention strategies that can reduce the effects of lead on hypertension. Additional studies are needed to confirm these findings in similar and more diverse populations and to assess interactions between environmental and psychosocial factors on other cardiovascular-related outcomes.

## Figures and Tables

**Table 1:** Characteristics, lead exposure levels, and economic status of subjects with stress measures by hypertension status at their first bone lead measurement.

Characteristic	Hypertensives (*n* = 276)	Nonhypertensives (*n* = 237)
Age (years)*	67.5 ± 6.8	66.2 ± 7.4
BMI (kg/m^2^)*	27.9 ± 3.7	26.8 ± 3.3
Family history of hypertension (%)^a^*		
Yes	45.8	27.4
No	54.2	72.6
Education (%)		
Less than high school	41.1	43.1
Graduated high school	58.9	56.9
Self-reported stress (%)		
High	21.4	21.5
Low	78.6	78.5
Sodium (mg/day)^b^	3,243 ± 939	3,404 ± 1,015
Potassium (mg/day)^b^	3,296 ± 640	3,315 ± 571
Calcium (mg/day)^b^*	804 ± 292	860 ± 310
Alcohol (g/day)	13.0 ± 16.2	14.0 ± 16.6
Smoking (pack-years)	19.2 ± 23.5	22.6 ± 23.9
Physical activity (kcal/week)	2,388 ± 1,708	2,638 ± 1,952
Trabecular lead (μg/g)	22.3 ± 14.6	20.5 ± 11.7
Patella lead (μg/g)	32.5 ± 20.1	30.3 ± 18.3
Blood lead (μg/dL)	6.3 ± 4.0	6.2 ± 4.2
SBP (mmHg)*	143.7 ± 16.7	124.0 ± 9.4
DBP (mmHg)*	84.8 ± 10.0	76.3 ± 6.5

Values are mean ± SD except where noted.

a History of physician-diagnosed hypertension in subject’s father or mother.

b Adjusted for total calorie intake.

* *p* < 0.05 for comparison between those with and without hypertension.

**Table 2:** Logistic regression of the effect of high stress on the relationship of tibia lead and patella lead on baseline hypertension status.

Covariate	OR^a^ (95% CI)
Model with tibia lead^b^	
Hypertensives	
High self-reported stress	1.05 (0.66 to 1.70)
Tibia lead	1.17 (0.88 to 1.42)
Tibia lead by high stress	1.00 (0.96 to 1.03)
Nonhypertensives	Referent
Model with patella lead^b^	
Hypertensives	
High self-reported stress	1.07 (0.66 to 1.73)
Patella lead	1.08 (0.85 to 1.38)
Patella lead by high stress	1.02 (0.99 to 1.05)
Nonhypertensives	Referent

a Odds ratios (ORs) based on 1-SD increase in tibia lead (11.6 μg/g) or patella lead (17.1 μg/g).

b Models adjusted for age and age squared; BMI; family history of hypertension; education; pack-years of smoking; alcohol intake; physical activity; and sodium, calcium, and potassium intake.

**Table 3:** Multiple regression of the effect of high stress on the relationship of patella lead and tibia lead on SBP and DBP.

	SBP		DBP	
Covariate	β	95% CI	β	95% CI
Model with tibia lead^a^				
High self-reported stress	2.89*	−0.16 to 5.94	−0.58	−2.69 to 1.52
Tibia lead	−0.27	−1.70 to 1.29	−0.30	−1.33 to 0.74
Tibia lead by high stress	3.77**	0.46 to 7.09	0.69	−1.60 to 2.98
Model with patella lead^a^				
High self-reported stress	2.98*	−0.12 to 6.08	−0.74	−2.86 to 1.38
Patella lead	0.02	−1.44 to 1.48	−0.59	−1.59 to 0.41
Patella lead by high stress	2.60	−0.95 to 6.15	−0.23	−2.65 to 2.20

a Parameter estimates are based on 1-SD increase in tibia lead (11.6 μg/g) or patella lead (17.1 μg/g). Models adjusted for age and age squared; BMI; family history of hypertension; education; pack-years of smoking; alcohol intake; physical activity; and sodium, calcium, and potassium intake.

* *p* < 0.10.

** *p* < 0.05.

**Table 4:** Cox proportional hazard regression of the effect of high stress on the relationship of patella lead and tibia lead on risk of hypertension.

Covariate	RR (95% CI)
Model with tibia lead^a^	
High self-reported stress	1.30 (0.77 to 2.19)
Tibia lead	0.89 (0.65 to 1.23)
Tibia lead by high stress	2.66 (1.43 to 4.95)*
Model with patella lead^a^	
High self-reported stress	1.48 (0.89 to 2.45)
Patella lead	1.18 (0.92 to 1.51)
Patella lead by high stress	2.64 (1.42 to 4.92)*

a Rate ratios are based on 1-SD in tibia lead (11.6 μg/g) or patella lead (17.1 μg/g). Model adjusted for age and age squared; BMI; family history of hypertension; education; pack-years of smoking; alcohol intake; physical activity; and sodium, calcium, and potassium intake, as well as baseline systolic blood pressure and diastolic blood pressure.

* *p* < 0.05
